# Supplementary material for: Constrained patterning of orientated metal chalcogenide nanowires and their growth mechanism
Source: Nat Commun. 2024 Jul 18;15:6074. doi: 10.1038/s41467-024-50525-4 (PMC11258352; doi:10.1038/s41467-024-50525-4)
Supplement: Supplementary file 1 — Supplementary Information [file 41467_2024_50525_MOESM1_ESM.pdf]

**Supplementary Information for**

# Constrained patterning of orientated metal chalcogenide nanowires and their growth mechanism

Qishuo Yang,[1,2,3] Yun-Peng Wang,[4] Xiao-Lei Shi,[5] XingXing Li,[1] Erding Zhao,[1] Zhi-Gang Chen,[5] Jin Zou,[6] Kai Leng,[7] Yongqing Cai,[8] Liang Zhu,[1]* Sokrates T. Pantelides,[9,10] Junhao Lin[1,2]*

[1]*Department of Physics and Shenzhen Key Laboratory of Advanced Quantum Functional Materials and Devices, Southern University of Science and Technology, Shenzhen 518055, People's Republic of China.*

[2]*Quantum Science Center of Guangdong-Hong Kong-Macao Greater Bay Area (Guangdong), Shenzhen 518045, People's Republic of China.*

[3]*School of Mechanical and Mining Engineering, The University of Queensland Brisbane, Queensland 4072, Australia.*

[4]*School of Physics and Electronics, Hunan Key Laboratory for Super-Micro Structure and Ultrafast Process, Central South University, Changsha 410083, People's Republic of China.*

[5]*School of Chemistry and Physics, Queensland University of Technology Brisbane, Queensland 4000, Australia.*

[6]*Center for Microscopy and Microanalysis, The University of Queensland Brisbane, St Lucia, Queensland 4072, Australia*

[7]*Department of Applied Physics, Hong Kong Polytechnic University, Hung Hom, Kowloon, Hong Kong, China.*

[8]*Institute of Applied Physics and Materials Engineering, University of Macau, Taipa, Macau SAR 999078, China.*

[9]*Department of Physics and Astronomy, Vanderbilt University, Nashville, Tennessee 37235, United States*

[10]*Department Electrical and Computer Engineering, Vanderbilt University, Nashville, Tennessee 37235, United States*

**Supplementary Note. 1 Sample preparation of transferring heterostructure onto microelectromechanical-system chips**

We use a fully automatic 2D materials transfer platform to pick up, stack, and transfer 2D material flakes on silicon wafers. The fabricated graphite-confined heterostructure on MEMS chip is shown in Supplementary Fig. 1a. The detailed sample preparation process is shown in Supplementary Fig. 2. First, using the incorporated optical microscope, we first align the centre of the PC-micro dome with the sample and slowly bring the Polycarbonate (PC) film close to the sample. After making contact, heat the stage to 130 °C to increase the adhesion force of the PC film and then lift-up the sample. After this, repeat this step to build the graphene-encapsulated $MoTe_2$ heterostructure. Next, attach the heterostructure to the surface of a MEMS chip and heat the stage to 180 °C to melt the PC. As the MEMS chip is very fragile, the entire process needs to be carried out very carefully and slowly. After the transfer is complete, immerse the chip in chloroform for 15 min to fully dissolve the PC.

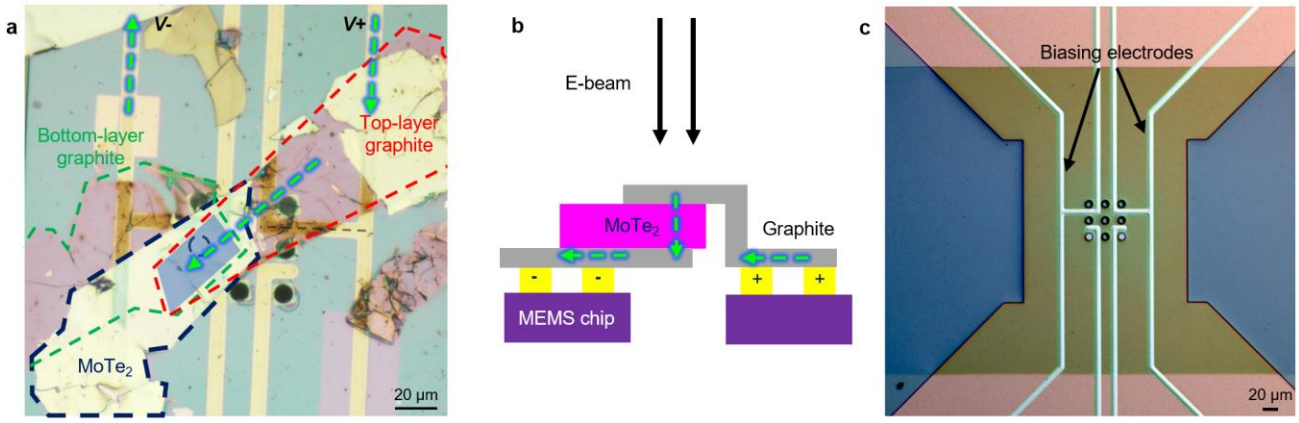


**Supplementary Fig. 1. Details related to the MEMS chip and sample preparation. a.** An optical image showcases the heterostructure positioned on the MEMS chip. The sections delineated by red and green dashed lines signify the upper and lower graphite layers, respectively. The specimen demarcated by blue dashed lines denotes the few-layers $MoTe_2$. The region shaded in blue designates the defined heterostructure area where $MoTe_2$ is covered by graphite on both sides. The device cross-sectional schematic is shown in **b**. **c.** Optical microscope images presenting the electrode positions (black arrows highlighted). The current path is highlighted by green dash line in **a-b**.

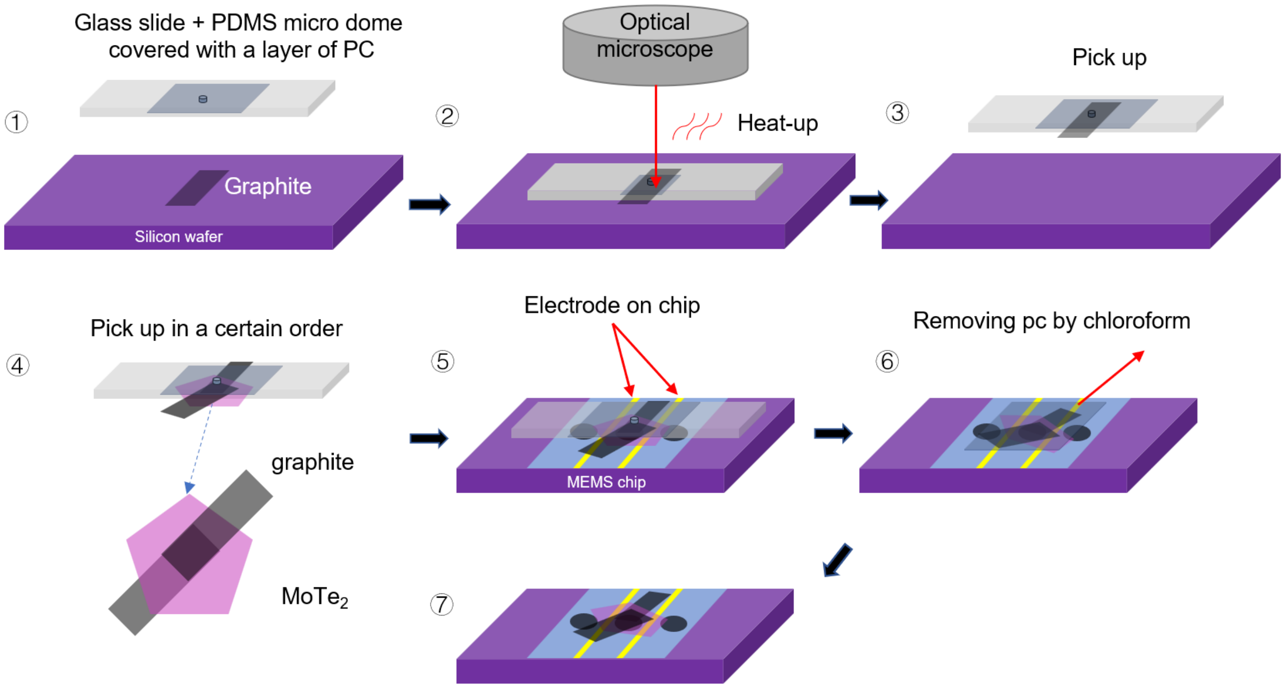


**Supplementary Fig. 2. 2D transfer method using polycarbonate (PC).** A PDMS micro-dome is used to support the PC film. Schematic ①-④ present the 2D heterostructure transfer process by picking up and stacking different 2D materials layers (graphite and $MoTe_2$ in this case) on the silicon wafer. Then the fabricated heterostructure is carefully attached and dropped down to the imaging region of the MEMS chip as shown in ⑤-⑦.

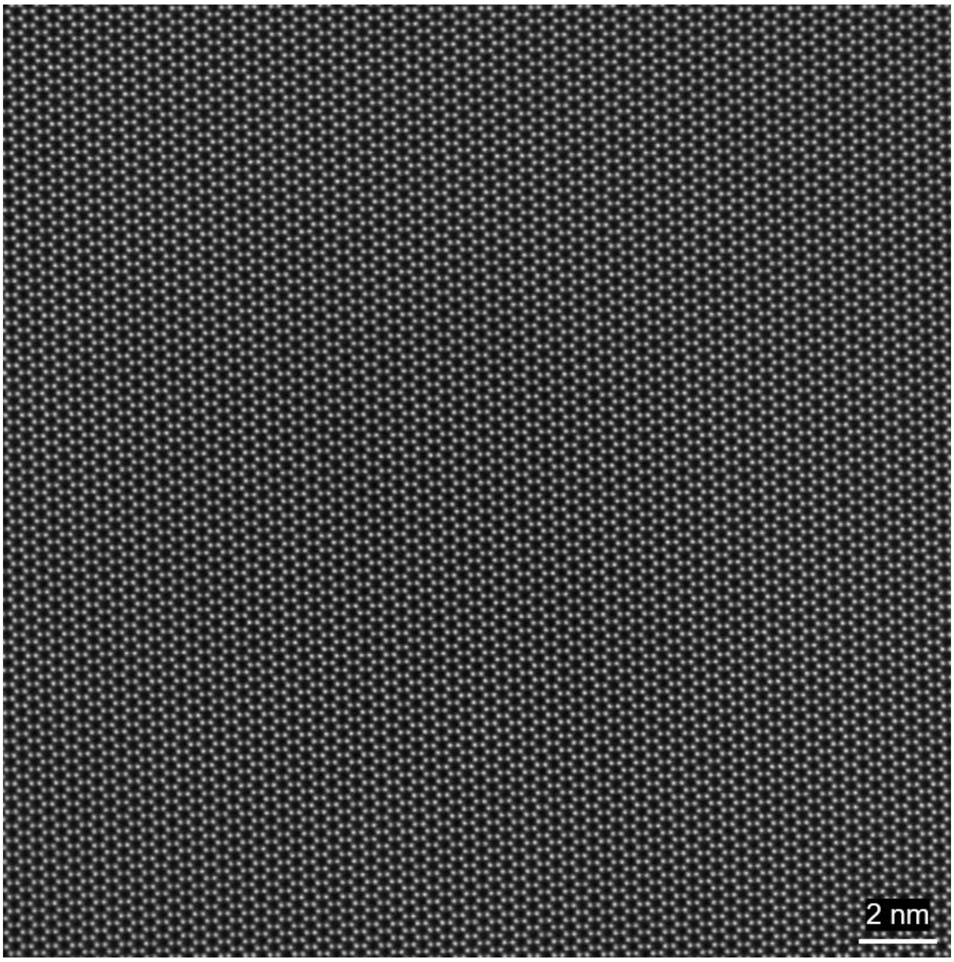


**Supplementary Fig. 3. High-magnification HAADF-STEM image of the 2H-$MoTe_2$.**

## Supplementary Note. 2 Preparation of the FIB sample on MEMS chips

To support the sample in the MEMS chips' free-standing hole, we first use silver glue to fill the chip. After the silver glue solidify, thick layer graphite is dropped down onto the surface of the sample as protection layer during FIB preparation.

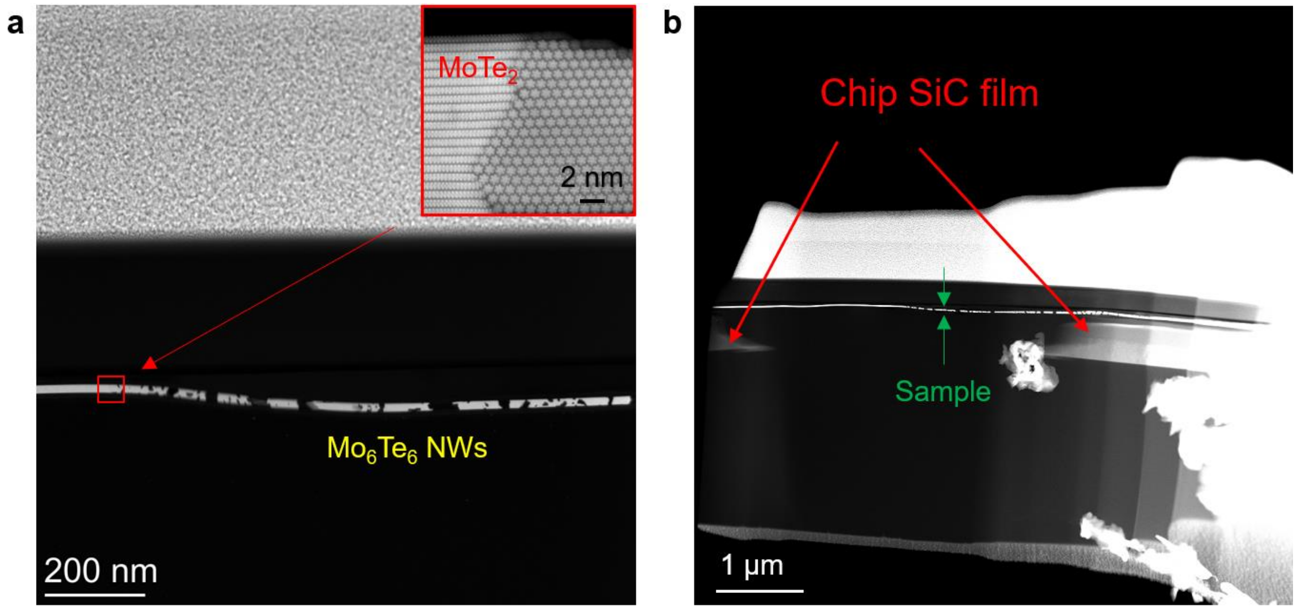


**Supplementary Fig. 4**. **Surface rippling of the heterostructure in the free-standing hole. a-b.** Cross-sectional HAADF-STEM images present the sample geometry. The cross-sectional interface between the 2H-$MoTe_2$ and $Mo_6Te_6$ NWs is shown in the upper-right in **a**.

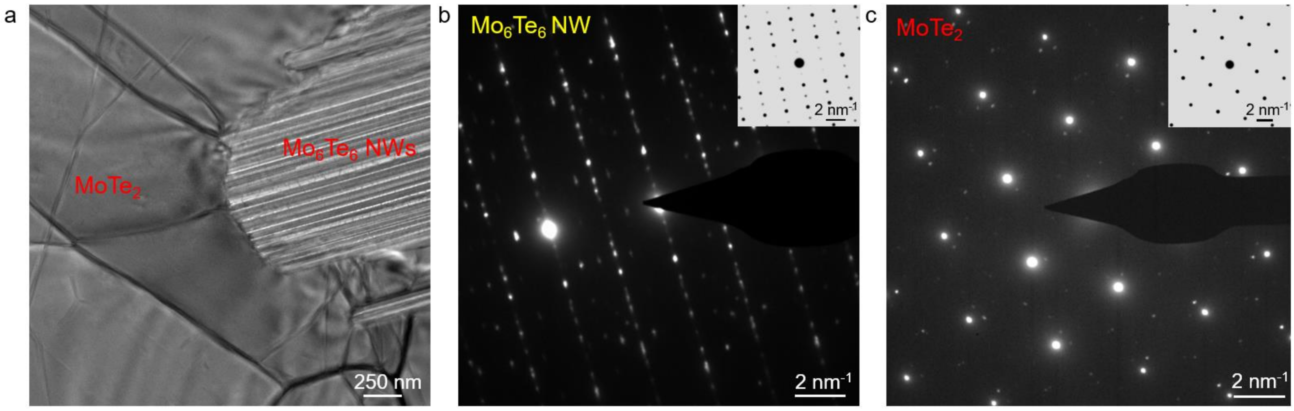


**Supplementary Fig. 5. Selected area electron diffraction (SAED) results for 2H $MoTe_2$ and $Mo_6Te_6$ nanowires (NWs). a.** TEM image highlighting the interface between 2H $MoTe_2$ and $Mo_6Te_6$ NWs. **b-c.** SAED patterns corresponding to NWs and $MoTe_2$ with simulated SAED pattern inserted.

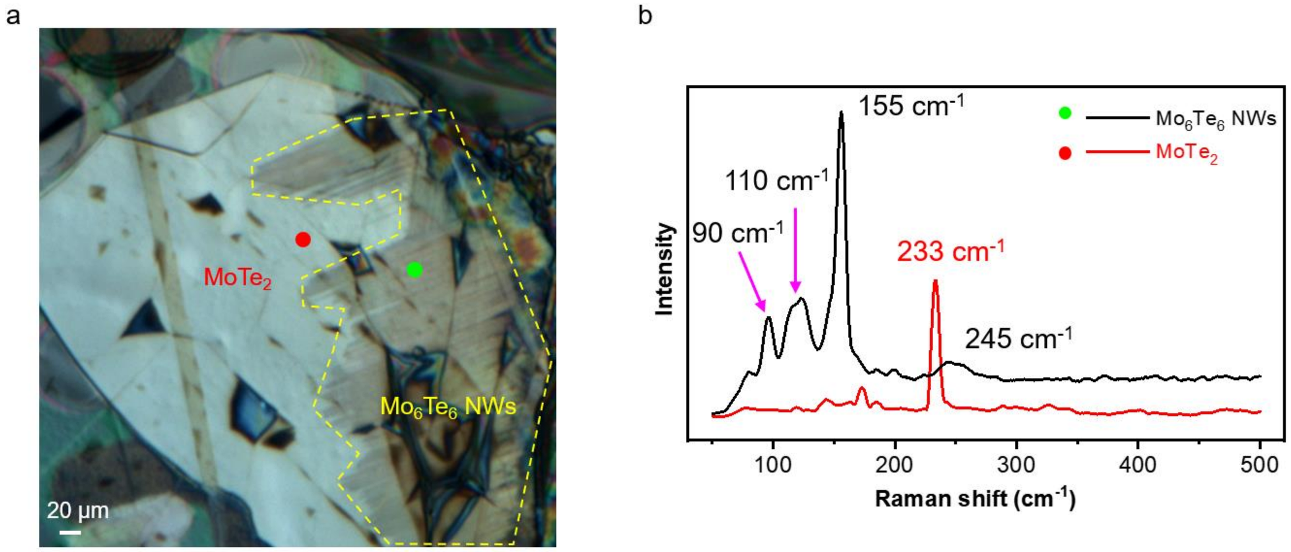


**Supplementary Fig. 6. Raman spectra for 2H $MoTe_2$ and $Mo_6Te_6$ NWs. a.** OM image showcases the sample used for acquiring Raman spectra. **b.** Raman spectra for $Mo_6Te_6$ NWs and $MoTe_2$, two peaks marked by purple arrows are newly found in our oriented NW.

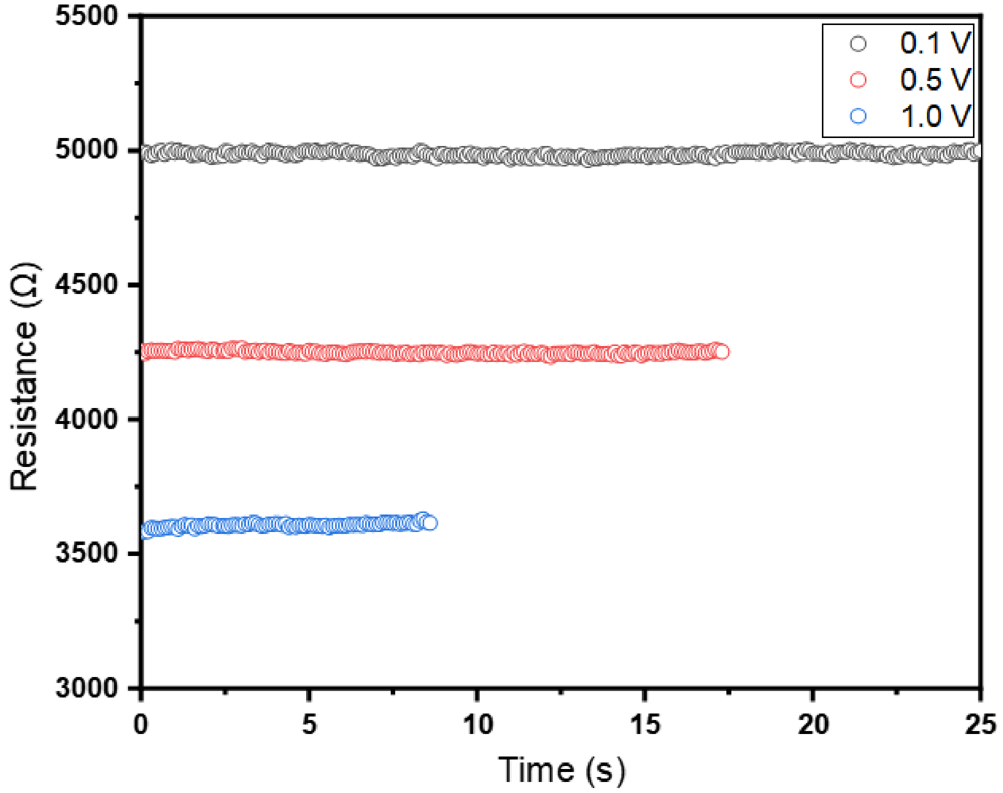


**Supplementary Fig. 7. The change in resistance before reaching the conversion threshold voltage.** *T*-*R* curve measured at 0.1 V, 0.5 V, and 1.0 V bias voltage. The resistance of the heterostructure decreases with increasing bias voltage due to carrier injection. The resistance remains constant when the bias voltage is stable, suggesting the absence of structural changes during this phase.

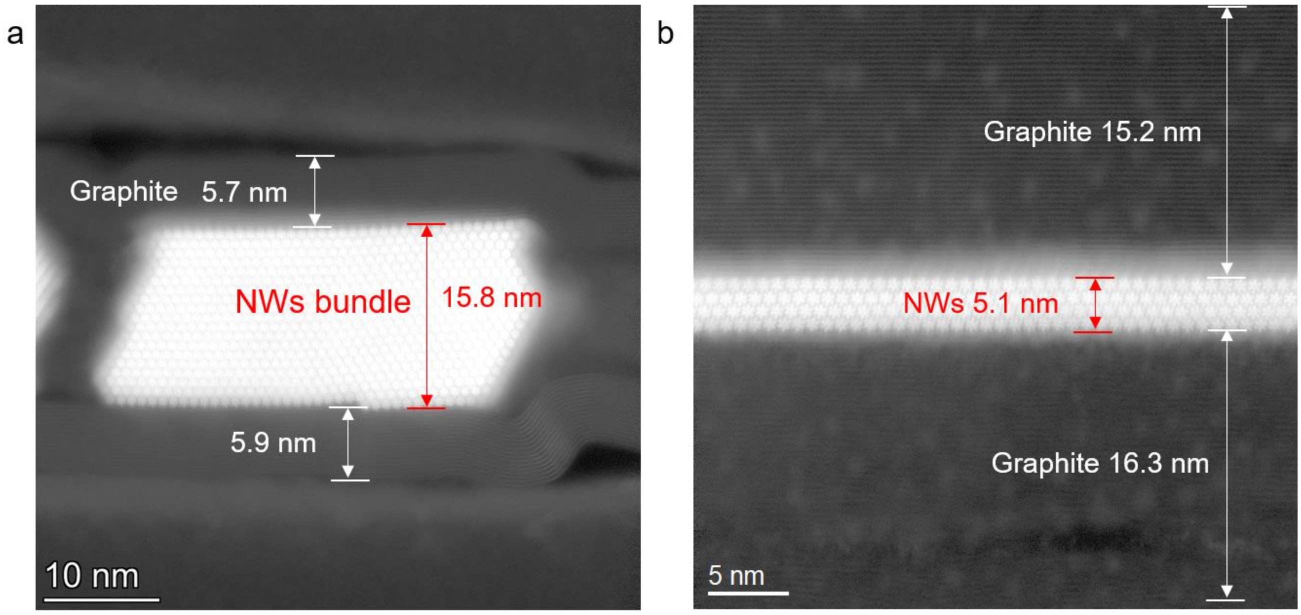


**Supplementary Fig. 8. The thickness of the graphite and $MoTe_2$ layers in the electric biasing experiment. a-b.** Cross-sectional high-angle annular dark field (HAADF) STEM images illustrating the converted NWs in two electric biasing experiments. The graphite layer thickness varies from 5.7 nm and 5.9 nm for the first biasing experiment and 15-16 nm for the second one.

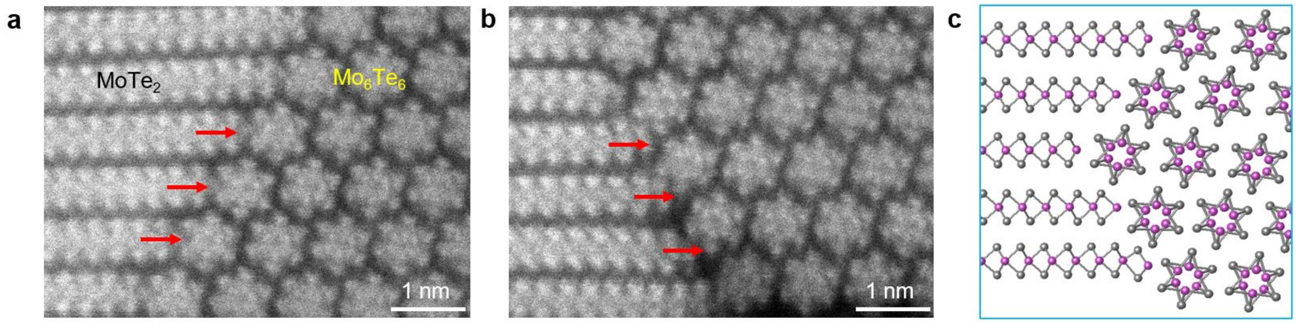


**Supplementary Fig. 9.** High-magnification cross-sectional STEM image of the interface between $MoTe_2$ and $Mo_6Te_6$ NWs. Matched **(a)** and mismatched **(b)** arrangement between 2H-$MoTe_2$ and $Mo_6Te_6$ NWs at the lateral interface. **c.** The corresponding lattice model.

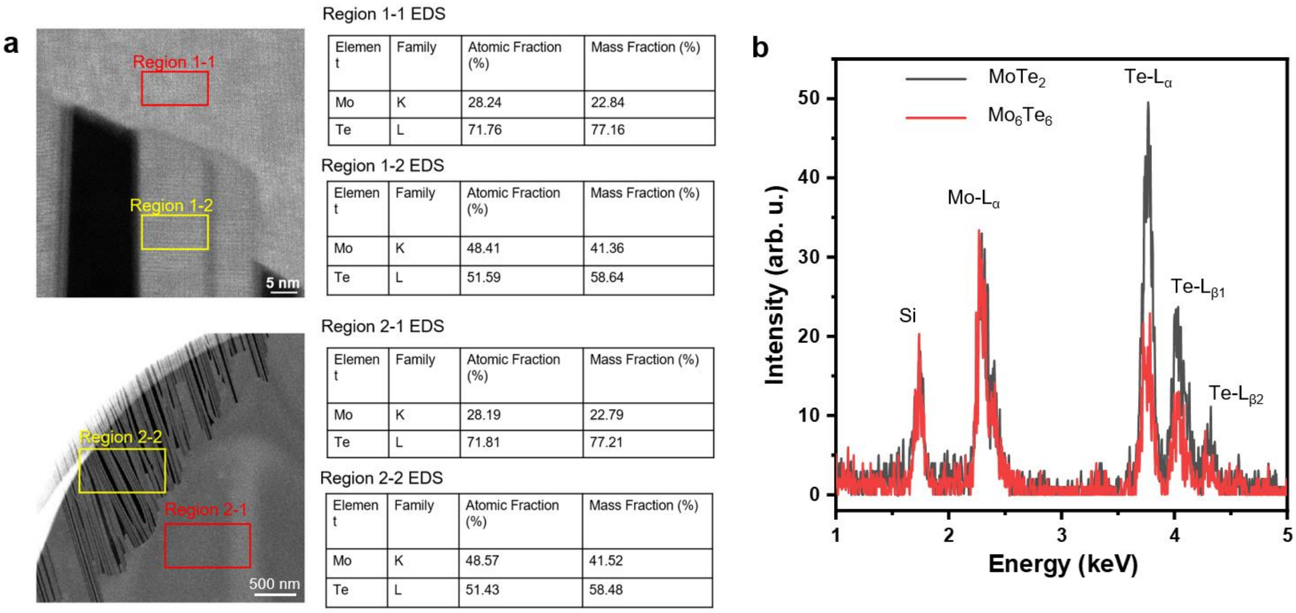


Region 1-1 EDS

| Element | Family | Atomic Fraction (%) | Mass Fraction (%) |
|---|---|---|---|
| Mo | K | 28.24 | 22.84 |
| Te | L | 71.76 | 77.16 |

Region 1-2 EDS

| Element | Family | Atomic Fraction (%) | Mass Fraction (%) |
|---|---|---|---|
| Mo | K | 48.41 | 41.36 |
| Te | L | 51.59 | 58.64 |

Region 2-1 EDS

| Element | Family | Atomic Fraction (%) | Mass Fraction (%) |
|---|---|---|---|
| Mo | K | 28.19 | 22.79 |
| Te | L | 71.81 | 77.21 |

Region 2-2 EDS

| Element | Family | Atomic Fraction (%) | Mass Fraction (%) |
|---|---|---|---|
| Mo | K | 48.57 | 41.52 |
| Te | L | 51.43 | 58.48 |

**Supplementary Fig. 10. EDS mapping of the selected $MoTe_2$ and $Mo_6Te_6$ NWs region. a.** Atomic ratio of the Mo and Te of the selected region in different frames. **b.** EDS analysis of the $MoTe_2$ and $Mo_6Te_6$ regions, a decrease in the Te peaks intensity in $Mo_6Te_6$ NWs can be observed.

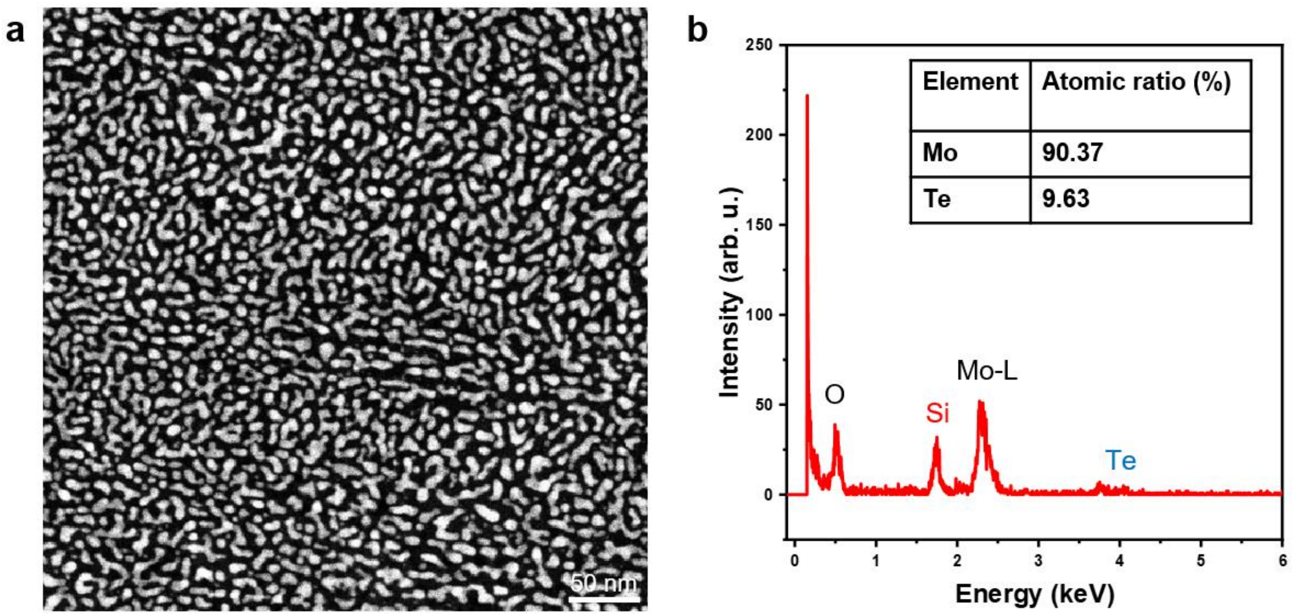


**Supplementary Fig. 11. EDS mapping of the selected amorphous film composed of clusters. a.** Low-magnification HAADF image presenting the morphology of the amorphous film. The original $MoTe_2$ in this hole is not fully graphite covered. **b.** EDS analysis reveals the atomic ratio of molybdenum (Mo) to tellurium (Te) in the amorphous Mo-clusters film, indicating a significant reduction in the concentration of Te atoms compared to pristine $MoTe_2$.

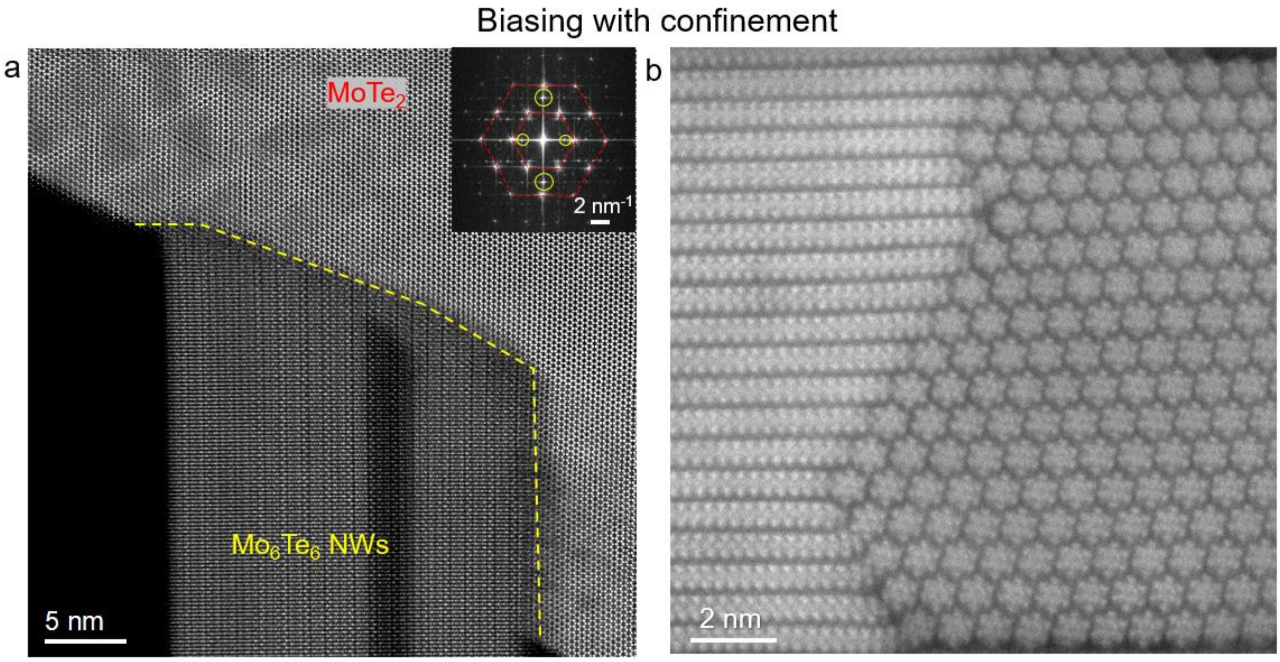


**Supplementary Fig. 12. The interface structure captured in the in-situ electric biasing experiment.** Atomic-resolution HAADF-STEM image showcasing the **(a)** top-view and **(b)** cross-sectional sharp interface, with the corresponding FFT pattern shown in the upper right in **a**.

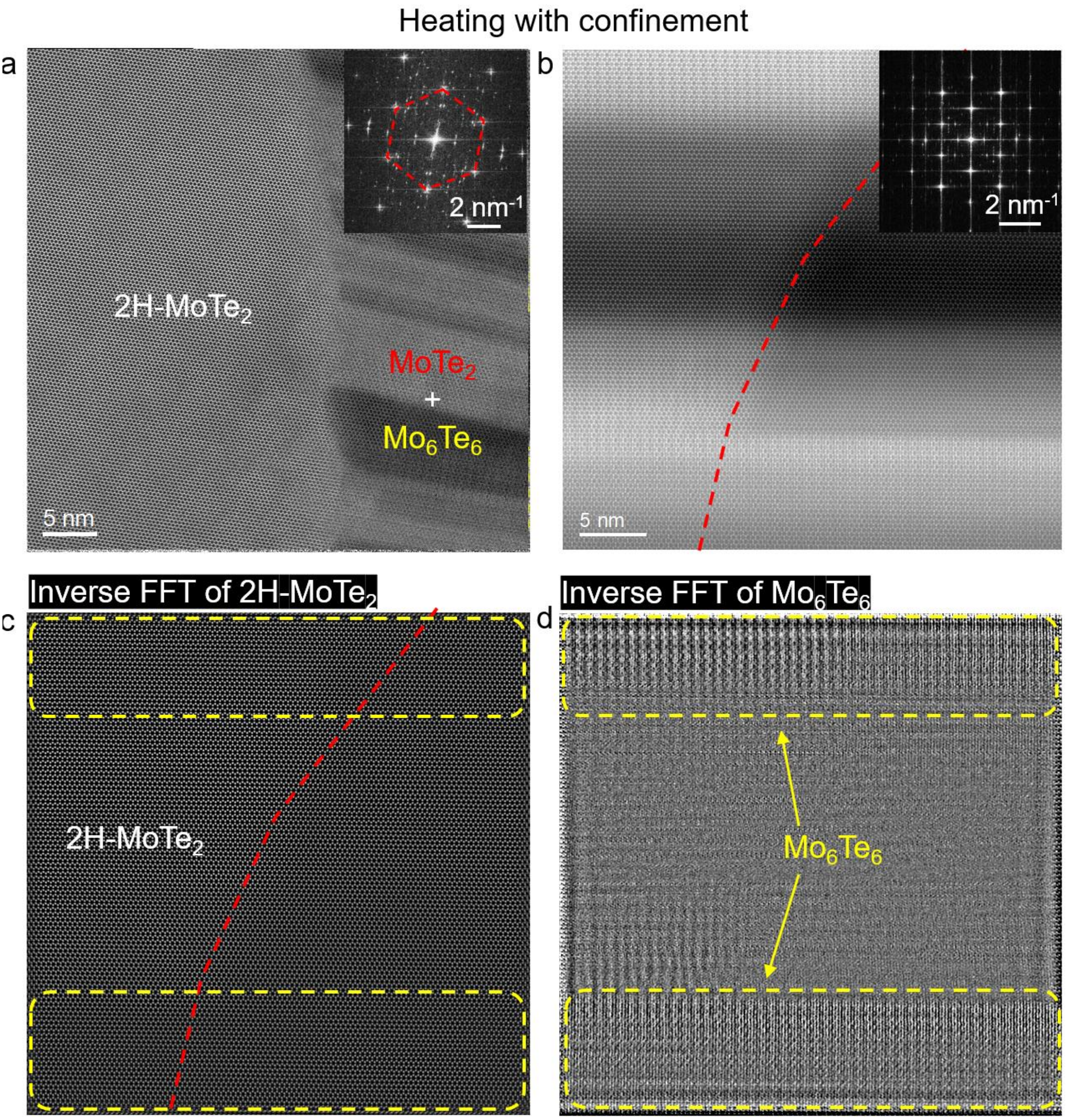


**Supplementary Fig. 13. The interface structure captured in the in-situ heating experiment.** a-b. High-resolution HAADF-STEM images offering a detailed view of the phase-overlapping at the $MoTe_2$-$Mo_6Te_6$ NW interfaces. Corresponding fast Fourier transform (FFT) images are shown in the upper right. c, d Inverted FFT analysis images using the $MoTe_2$ and $Mo_6Te_6$ patterns in b, conclusively confirming the observed phase-overlapping.

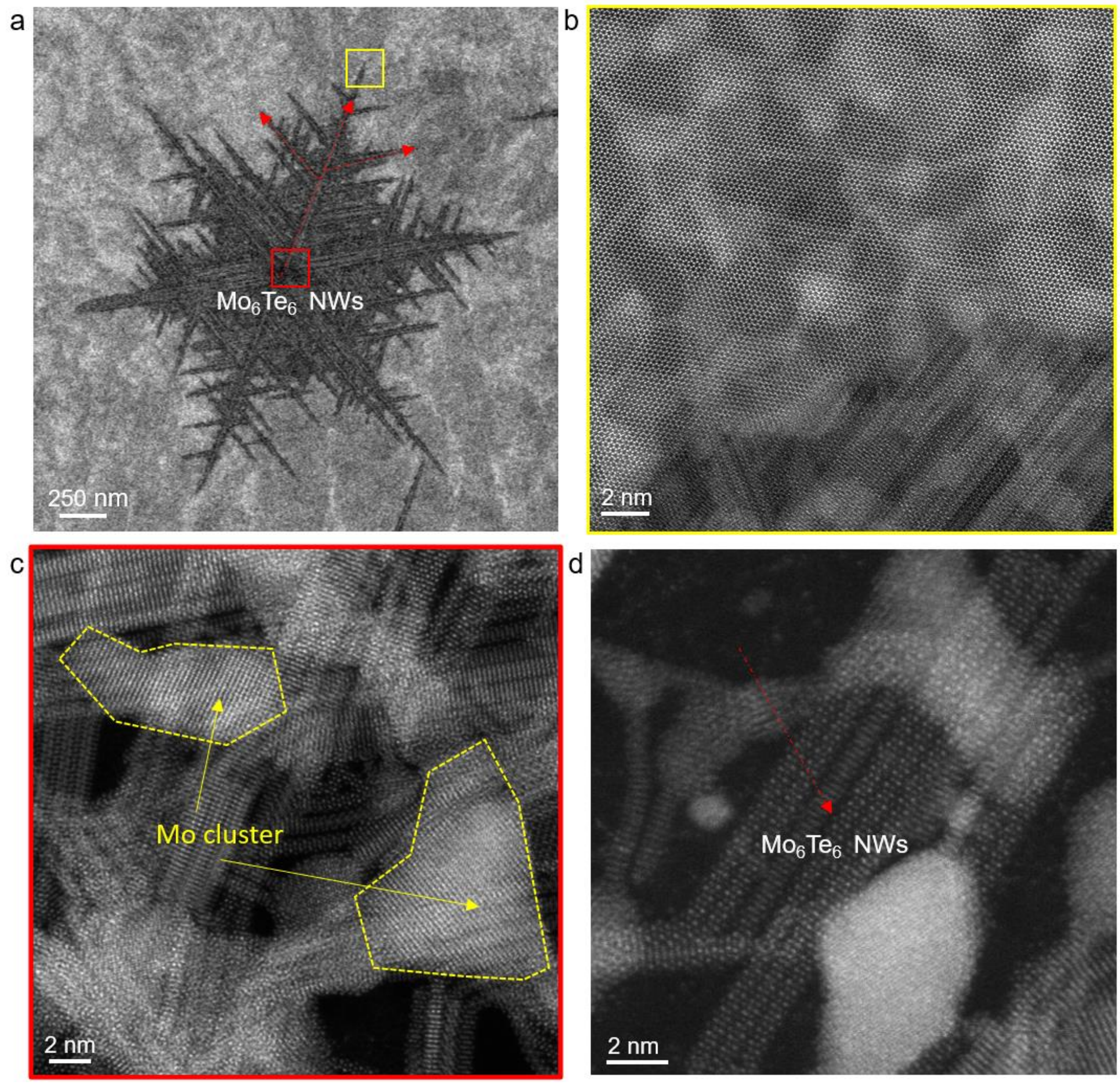


**Supplementary Fig. 14. Interface structure captured in in-situ heating experiment without graphite confinement. a.** low-magnification STEM image shows the converted snow-flake-like $Mo_6Te_6$NWs. **b.** atomic-resolution STEM image taken at the growth frontier of NW bundles (yellow square highlighted in **a**). **(c)** and **(d)** are taken at the nucleation point highlighted by a red square in **a**. Random size Mo clusters and disordered NWs can be found in **b-d**.

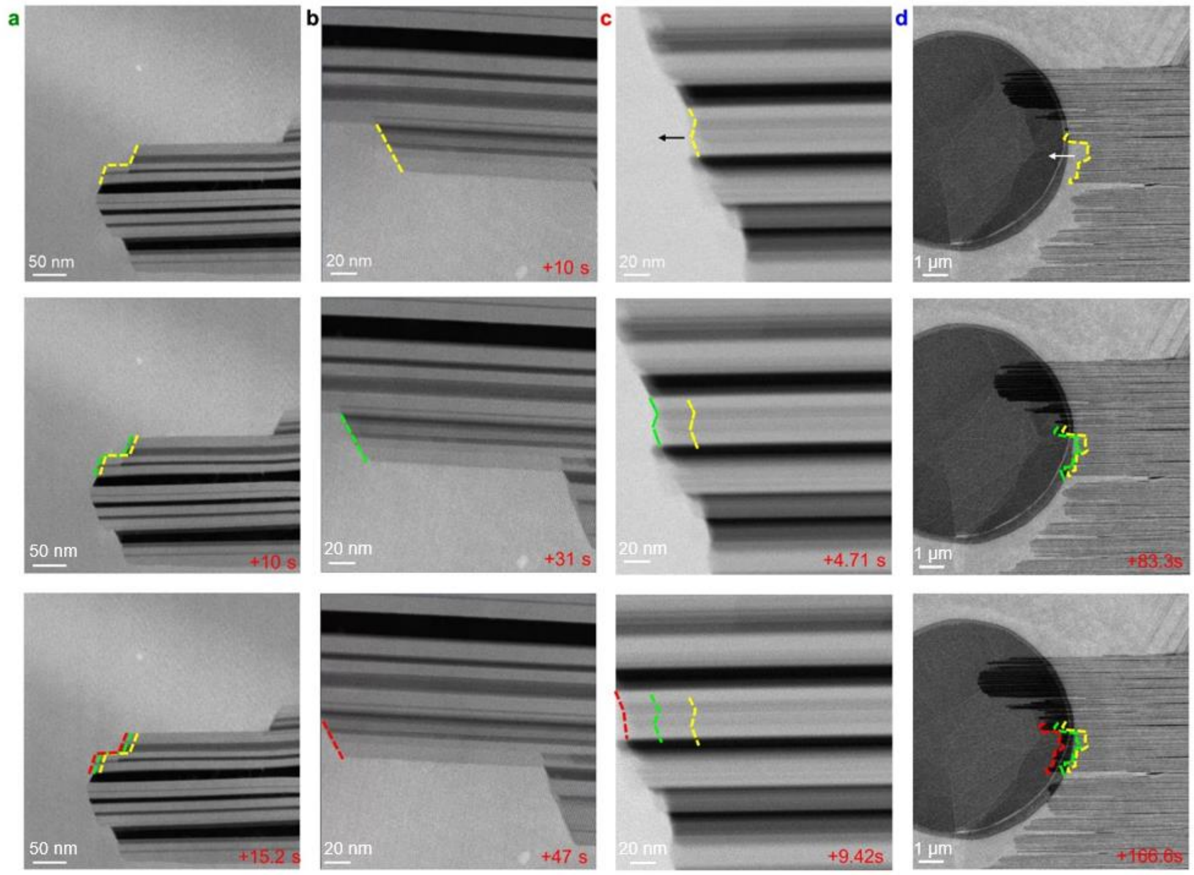


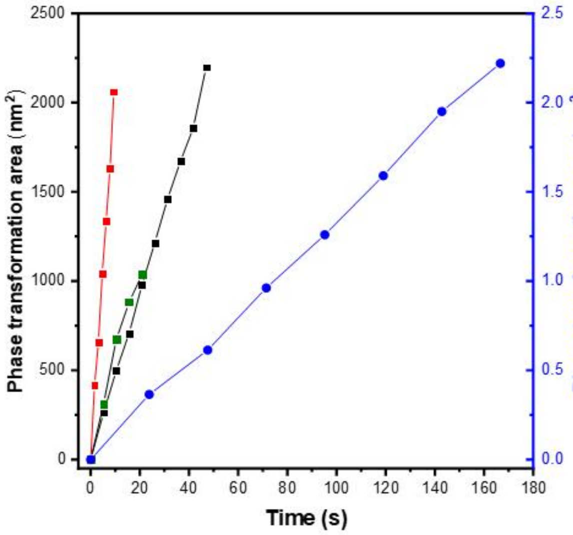


| | Transformation rate | Growth speed |
|---|---|---|
| 3 V biasing (a) | 50.7 $nm^2s^{-1}$ | 1.02 nm $s^{-1}$ |
| 4 V biasing (b) | 47.5 $nm^2s^{-1}$ | 1.28 nm $s^{-1}$ |
| 650 °C heating (c) | 208 $nm^2s^{-1}$ | 5.91 nm $s^{-1}$ |
| 700 °C heating (d) | 13300 $nm^2s^{-1}$ | 6.48 nm $s^{-1}$ |

**Supplementary Fig. 15. Phase transformation area vs time in the biasing and heating experiments.** HAADF-STEM presenting the phase transformation area vs time in **(a-b)** electrical biasing, and **(c-d)** heating experiments. The phase transformation area vs time are shown in **e**. **f.** The statistical data regarding the transformation and growth rate. The scale for curve **d** (blue curve in **e**) is $\mu m^2$. (see **Supplementary Movies 1, 3-5**)

**Supplementary Note. 3 The Nucleation of $Mo_6Te_6$ NWs**

Nucleation of $Mo_6Te_6$ NWs start from the edge at the lower graphite-$MoTe_2$ interface, and rapidly convert adjacent vertical-stacked $MoTe_2$ layers into NWs while expanding much slower in the horizontal direction. The rapid vertical growth is enabled by the two factors: 1) the NWs are metallic so that the resistive Schottky junction moves rapidly with the NWs higher up and 2) the Te atoms that are released by the Joule heating can quickly fly out of the sandwich at the edge. During the horizontal growth, however, Te atoms need to navigate their way out.

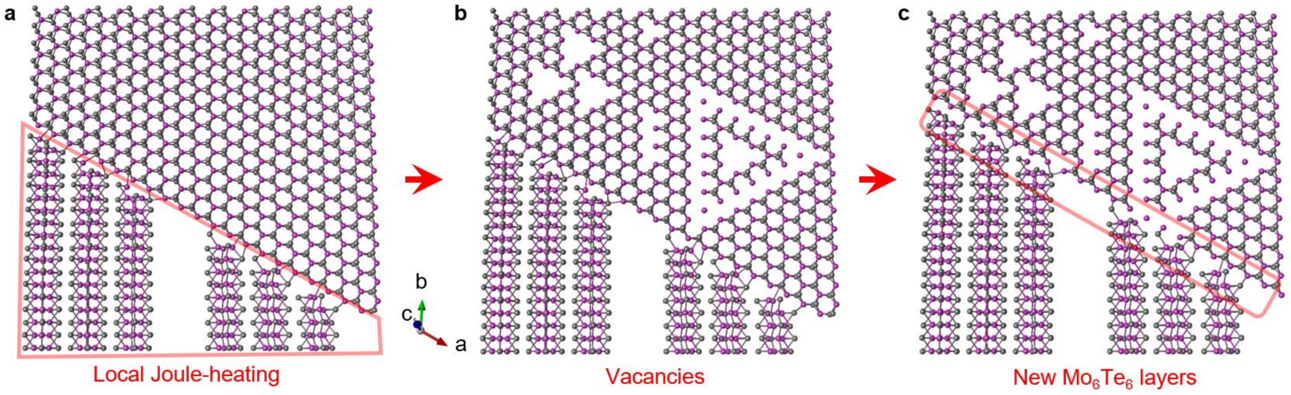


**Supplementary Fig. 16. Schematic of the $Mo_6Te_6$ NWs growth along one specific zigzag edge direction. a-c.** Bias voltage generates local Joule-heating near the interface, which further induces Te vacancies in the $MoTe_2$ region that leads to the generation of new layers of NWs at the interface.

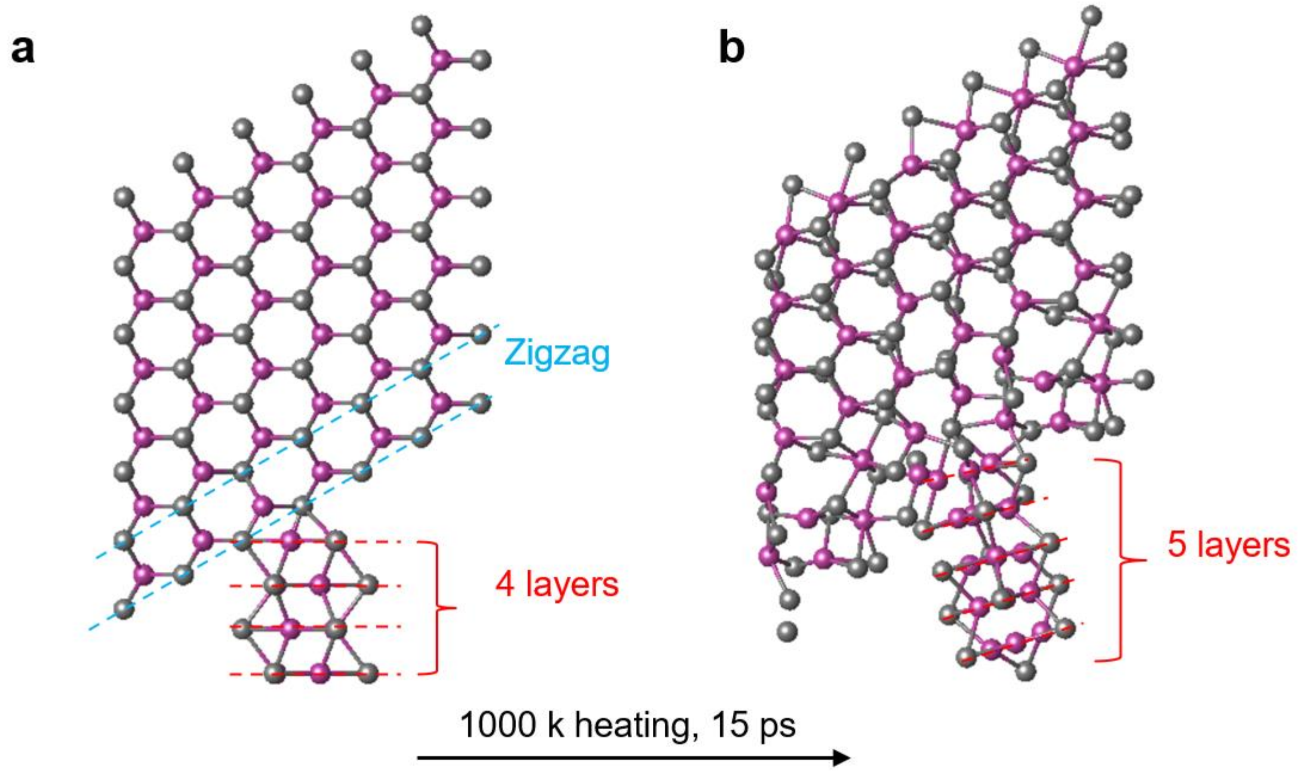


**Supplementary Fig. 17. Molecular dynamics (MD) simulations of a single NW attach to a zigzag edge of $MoTe_2$**. Before simulation (**a**) and after simulation (**b**), the placed NW undergoes rotation and finally attaches to the zigzag edge of the $MoTe_2$. When we placed a single NW facing a slanted zig-zag edge, the NW twisted perpendicular to the edge to bond. To avoid the twisting, the NW was placed as shown in Fig. 5b(left).

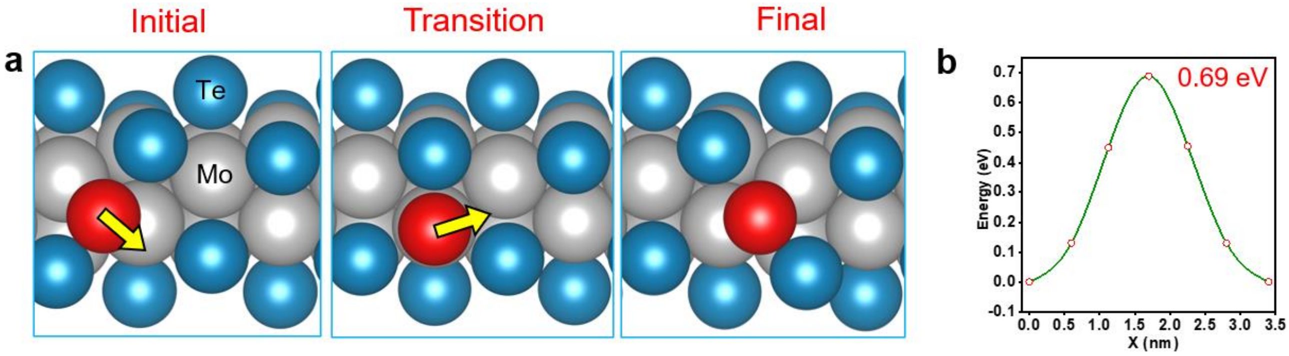


**Supplementary Fig. 18. DFT calculations of a Te atom straddling a $Mo_3Te_3$ ring on a single NW with 0.69 eV energy barrier. a**. schematic showcasing a Te atom (red highlighted) straddling a $Mo_3Te_3$ ring, the migration paths are highlighted by yellow arrows. The calculated migration barrier is shown in **b**.

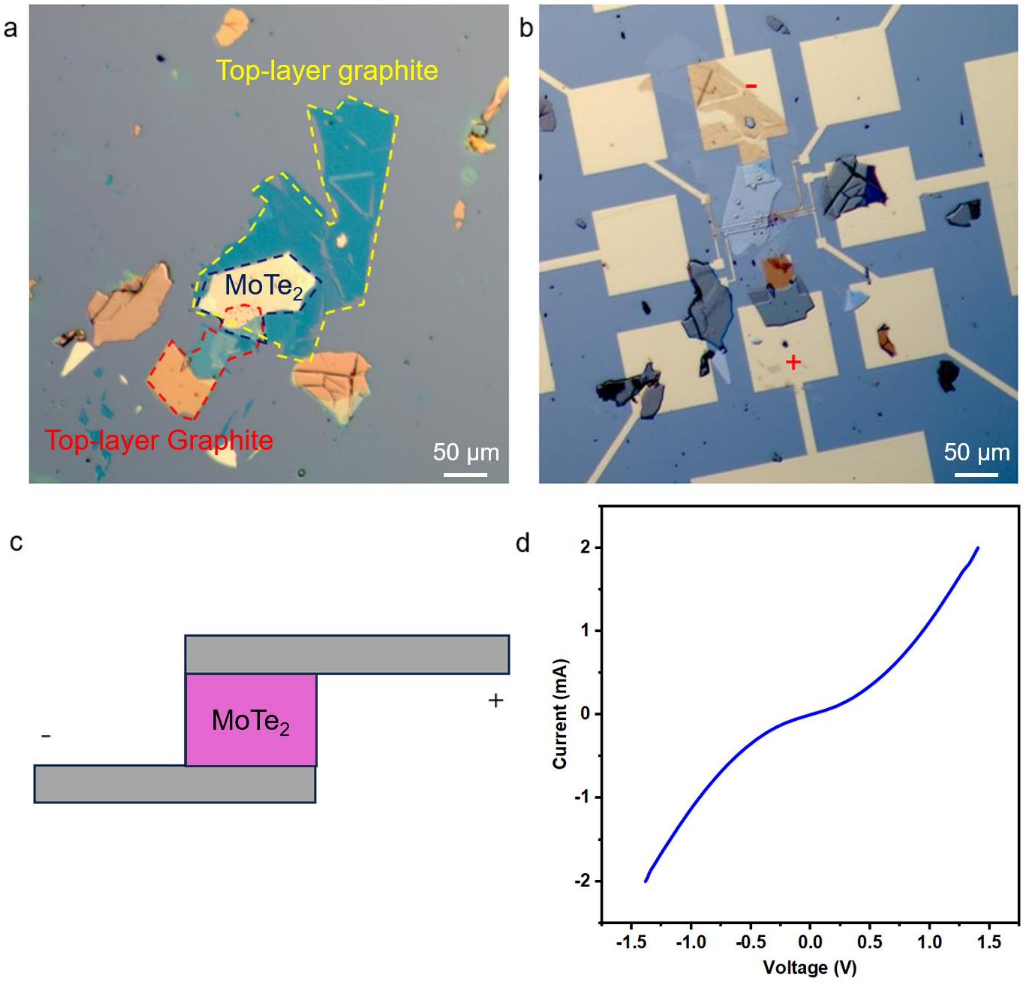


**Supplementary Fig. 19. The transport results of the graphite confined $MoTe_2$.** Optical microscope images of the **(a)** fabricated graphite confined $MoTe_2$ and **(b)** fabricated device on silicon wafer. **c.** cross-sectional schematic diagram of the device. **d.** I-V curve measured from the device.

| References | Phase transition | Carrier motilities ($cm^2\ V^{-1}\ s^{-1}$) | Schottky barrier height (meV) | Contact resistance (Ω μm) | Direct phase patterning control |
|---|---|---|---|---|---|
| ***Adv. Funct. Mater.*** 2022, 32 (41), 2205299.[1] | 1T'/2H/1T' | 5.6 | 37 | $7.1\times10^3$ | Yes |
| ***Nat. Nanotechnol.*** 2017, 12 (11), 1064-1070.[2] | 1T'/2H/1T' | 16.2 | 25 | $1.4\times10^4$ | No |
| ***ACS Nano*** 2019, 13 (7), 8035-8046.[3] | 1T'/2H/1T' | 7~8 | 30±10 | 235 (1T')<br>$7.8\times10^6$ (2H) | No |
| ***Nano Lett.*** 2019, 19 (10), 6845-6852.[4] | 1T'/2H/1T' | - | 23 | $1.1\times10^3$ | Yes |
| ***Science*** 2015, 349 (6248), 625-628.[5] | 2H-1T' | 50 | ~10 (for 1T'), ~200 (for 2H) | ~$1\times10^3$ (2H contact)<br>~100 (1T' contact) | Yes |
| ***Adv. Mater.*** 2017, 29 (16), 1605461.[6] | 2H-1T' | - | - | - | No |
| ***ACS Appl. Nano Mater.*** 2020, 3, 10, 10411–10417.[7] | 2H-1T' | 15 | - | $3.64\times10^4$ (1T'/2H)<br>$8\times10^3$ (1T') | Yes |
| ***Science***, 372, 195-200 (2021).[8] | 1T'-2H | 45 | 6.15 (Vg=-55V) | $1.6\times10^3$ (1T') | No |
| ***Nano Lett.*** 2018, 18 (2), 675-681.[9] | $Mo_6Te_6$ Nanowires | - | - | - | No |
| ***Adv. Mater.*** 2017, 29 (18), 1606264.[10] | 2H→$Mo_6Te_6$ Nanowires | - | - | - | No |
| ***ACS Nano*** 2019, 13 (1), 642-648.[11] | $Mo_6Te_6$ Nanowires | 1139 | 8.7 | $2.85\times10^7$ | No |
| **This work** | 2H→$Mo_6Te_6$ Nanowires | 5.99 | 11.52 | 43.73 | Yes |

**Supplementary Table. 1. Comparisons of the contact resistance of 1T'/NWs phase and the performance of our NWs/2H/NWs FETs with other results reported in the literature (Suppl. refs. [1-11]).**

**Supplementary references:**


1. Zhang, S., et al. Field Effect Transistor Sensors Based on In-Plane 1T′/2H/1T′ $MoTe_2$ Heterophases with Superior Sensitivity and Output Signals. *Advanced Functional Materials*, **32,** 2205299 (2022)
2. Sung, J.H., et al. Coplanar semiconductor–metal circuitry defined on few-layer $MoTe_2$ via polymorphic heteroepitaxy. *Nature Nanotechnology*, **12,** 1064-1070 (2017)
3. Ma, R., et al. $MoTe_2$ Lateral Homojunction Field-Effect Transistors Fabricated using Flux-Controlled Phase Engineering. *ACS Nano*, **13,** 8035-8046 (2019)
4. Xu, X., et al. Scaling-up Atomically Thin Coplanar Semiconductor–Metal Circuitry via Phase Engineered Chemical Assembly. *Nano Letters*, **19,** 6845-6852 (2019)
5. Cho, S., et al. Phase patterning for ohmic homojunction contact in $MoTe_2$. *Science*, **349,** 625-628 (2015)
6. Yoo, Y., DeGregorio, Z.P., Su, Y., Koester, S.J., Johns, J.E. In-Plane 2H-1T′ $MoTe_2$ Homojunctions Synthesized by Flux-Controlled Phase Engineering. *Advanced Materials*, **29,** 1605461 (2017)
7. Yang, S., et al. Large-Scale Vertical 1T′/2H $MoTe_2$ Nanosheet-Based Heterostructures for Low Contact Resistance Transistors. *ACS Applied Nano Materials*, **3,** 10411-10417 (2020)
8. Xu, X., et al. Seeded 2D epitaxy of large-area single-crystal films of the van der Waals semiconductor 2H $MoTe_2$. *Science*, **372,** 195-200 (2021)
9. Yu, Y., Wang, G., Tan, Y., Wu, N., Zhang, X.-A., Qin, S. Phase-Controlled Growth of One-Dimensional $Mo_6Te_6$ Nanowires and Two-Dimensional $MoTe_2$ Ultrathin Films Heterostructures. *Nano Letters*, **18,** 675-681 (2018)
10. Zhu, H., et al. New $Mo_6Te_6$ Sub-Nanometer-Diameter Nanowire Phase from 2H-$MoTe_2$. *Advanced Materials*, **29,** 1606264 (2017)
11. Lee, R.S., Kim, D., Pawar, S.A., Kim, T., Shin, J.C., Kang, S.-W. van der Waals Epitaxy of High-Mobility Polymorphic Structure of $Mo_6Te_6$ Nanoplates/$MoTe_2$ Atomic Layers with Low Schottky Barrier Height. *ACS Nano*, **13,** 642-648 (2019)
