# Supplementary material for: Constrained patterning of orientated metal chalcogenide nanowires and their growth mechanism
Source: Nat Commun. 2024 Jul 18;15:6074. doi: 10.1038/s41467-024-50525-4 (PMC11258352; doi:10.1038/s41467-024-50525-4)
Supplement: Supplementary file 3 — Description of Additional Supplementary Files [file 41467_2024_50525_MOESM3_ESM.pdf]

Description of Additional Supplementary Files

File Name: Supplementary Movie 1

Description: Low-magnification HAADF-STEM movie taken during the conversion of 2H-$MoTe_2$ into Mo_6Te_6 NWs in biasing with graphite confinement.

File Name: Supplementary Movie 2

Description: Atomic-scale HAADF-STEM movie presents the dynamic facet evolution of 2H-$MoTe_2$ vs. the growth direction of Mo_6Te_6 NWs.

File Name: Supplementary Movie 3

Description: Low-mag HAADF-STEM movie taken during the conversion of 2H-$MoTe_2$ into Mo_6Te_6 NWs in heating with graphite confinement at 650℃.

File Name: Supplementary Movie 4

Description: Large-scale HAADF-STEM movie taken during the conversion of 2H-$MoTe_2$ into Mo_6Te_6 NWs in heating with graphite confinement at 700℃.

File Name: Supplementary Movie 5

Description: Large-scale HAADF-STEM movie taken during the conversion of 2H-$MoTe_2$ into Mo_6Te_6 NWs in biasing with graphite confinement.
